# Supplementary material for: Barriers to effective hypertension management in rural Bihar, India: A cross-sectional, linked supply- and demand-side study
Source: PLOS Glob Public Health. 2022 Oct 12;2(10):e0000513. doi: 10.1371/journal.pgph.0000513 (PMC10021531; doi:10.1371/journal.pgph.0000513)
Supplement: S1 Annex — Provides motivations and justifications for parent study sample size. (DOCX) [file pgph.0000513.s001.docx]

## S1 Annex: Assessment of Primary Health Care in Bihar Sample Size Calculations

**Sample size calculation to detect differences in care seeking preferences among households**

Stata code: power twoprop .5, diff(.05 .1)

Estimated sample sizes for a two-sample proportions test

Pearson's chi-squared test

Ho: p2 = p1 versus Ha: p2 != p1

| Alpha | Power | N | N1 | N2 | Delta | P1 | P2 | Difference |
| --- | --- | --- | --- | --- | --- | --- | --- | --- |
| .05 | .8 | 3130 | 1565 | 1565 | .05 | .5 | .55 | .05 |
| **.05** | **.8** | **776** | **388** | **388** | **.1** | **.5.** | **6** | **.1** |

776 * 2 (design effect) = 1,552

1,552 / 0.66 (percent of sick patients who sought care) = 2,351.5

2,352 / 0.06 (percent of individuals who were sick in last two weeks) = 39,191.6 individuals

39,191.6 / 5 (average household size) = 7,838.3 households

7,838.3 / 0.8 (20% non-response rate) = 9,797.9 households

**Sample size calculation to detect differences in quality across public and private providers**

Stata code: power twomeans 57, sd(7.25) diff(5(5)15)

Estimated sample sizes for a two-sample means test

t test assuming sd1 = sd2 = sd

Ho: m2 = m1 versus Ha: m2 != m1

| Alpha | Power | N | N1 | N2 | Delta | M1 | M2 | Difference | Standard Deviation |
| --- | --- | --- | --- | --- | --- | --- | --- | --- | --- |
| **.05** | **.8** | **68** | **34** | **34** | **5** | **57** | **62** | **5** | **7.25** |
| .05 | .8 | 20 | 10 | 10 | 10 | 57 | 67 | 10 | 7.25 |
| .05 | .8 | 10 | 5 | 5 | 15 | 57 | 72 | 15 | 7.25 |

34 public facilities and 34 private facilities required

35 * 1.5 (DEFT) = 52.5

52.5 / 0.8 (20% non-response rate) = 65.6 PHCs
