# Supplementary material for: Barriers to effective hypertension management in rural Bihar, India: A cross-sectional, linked supply- and demand-side study
Source: PLOS Glob Public Health. 2022 Oct 12;2(10):e0000513. doi: 10.1371/journal.pgph.0000513 (PMC10021531; doi:10.1371/journal.pgph.0000513)
Supplement: S2 Annex — Provides definitions and data sources for steps along the provider quality cascade. (DOCX) [file pgph.0000513.s002.docx]

## S2 Annex: Provider quality cascade

| **Traditional Cascade Step** | **Provider Cascade Step** | **Calculation** | **Data Source** |
| --- | --- | --- | --- |
| *Denominator* | Total number of providers | Number of providers included in provider survey | Provider assessment (all modules) |
|  | Providers who would treat hypertension | Providers who responded “Yes” to the question “Would you treat a 65-year old woman presenting to your clinic with a fever and reporting a previous BP measurement from a health worker?” | Hypertension vignette |
| Screened | Providers with a functioning BP measurement device | Providers who would treat hypertension and had a blood pressure measuring device (manual or digital) that was observed and functional on the day of the assessment | Facility assessment |
|  | Providers who would check a patient’s BP once | Providers with a functioning BP measurement device who stated that they would measure the hypothetical patient’s blood pressure | Hypertension vignette |
| Aware | Providers who make a correct htn diagnosis | Providers who checked the hypothetical patient’s BP and correctly diagnosed the hypothetical patient as having hypertension after hearing that the patient’s BP was 150/90 mmHg | Hypertension vignette |
| Treated | Providers who initiate treatment upon diagnosis | Providers who accurately diagnosed the hypothetical patient and stated that they would either “treat the person at the clinic without referral” or “refer the person and start some treatment at the clinic” | Hypertension vignette |
|  | Providers with at least one antihypertensive drug available | Providers who would start treatment on a diagnosed hypertensive patient and had least one of the following first line anti-hypertensive medicines available on the day of the assessment: amlodipine, hydrochlorothiazide, and atenolol | Facility assessment |
| Controlled | Providers who write an appropriate prescription | Providers with a first line anti-hypertensive drug available who would start treatment on a diagnosed hypothetical patient and wrote a prescription that would safely lower the patient’s BP | Hypertension vignette |
