# Supplementary material for: Barriers to effective hypertension management in rural Bihar, India: A cross-sectional, linked supply- and demand-side study
Source: PLOS Glob Public Health. 2022 Oct 12;2(10):e0000513. doi: 10.1371/journal.pgph.0000513 (PMC10021531; doi:10.1371/journal.pgph.0000513)
Supplement: S3 Annex — Provides the process undertaken by two clinicians for rating the appropriateness of prescriptions. (DOCX) [file pgph.0000513.s003.docx]

## S3 Annex: Prescription Rating Protocol for Clinical Vignettes

1. Prescriptions will be classified into three different categories:

- 1. Appropriate: Correct type of medication was prescribed as indicated, with appropriate dosage, frequency, and duration.
  2. Inappropriate not harmful: Prescription was missing one or more key medications as indicated by the primary diagnosis.
  3. Inappropriate and harmful: If one or more medication in the prescription may directly cause harm in patients, breastfed infants, or fetus based on the type of medication and/ or dosage.

2. Prescriptions will be rated as a whole, rather than individual drugs. (i.e. if one medication is classified harmful, then complete prescription will be marked as harmful)

3. Incomplete prescription will be marked as inappropriate. (e.g. ORS not prescribed in diarrhea)

4. Reviewer, to the best of their ability, will search for the generic analog of any brand names prescribed and will rate each medication based on their generic component/ active ingredients.

5. Homeopathic medications not recognized by the reviewer will be highlighted in yellow and will be excluded from the rating.

6. If the medication is not prescribed for the main diagnosis (e.g. hypertension) and the secondary issue is taken care of (e.g. headache), therefore the prescription will be marked as inappropriate

7. Drug appropriateness with diagnosis will be rated based on the primary diagnosis of the clinical vignettes and patient observation, differential diagnoses will be disregarded. For each relevant cases, the below clinical guidelines may be used:

- 1. Diarrhea in children: WHO Integrated Management of Childhood Illness (IMCI)^1^, <https://apps.who.int/iris/bitstream/handle/10665/104772/9789241506823_Chartbook_eng.pdf?sequence=16>
  2. Acute respiratory infection (ARI) with fever in children: WHO Integrated Management of Childhood Illness (IMCI)^1^, <https://apps.who.int/iris/bitstream/handle/10665/104772/9789241506823_Chartbook_eng.pdf?sequence=16>
  3. Angina pectoris in adult: American Heart Association’s Advance Cardiac Life Support (pre-hospital section).
  4. Adult hypertension: India’s Standard Treatment Guideline of Hypertension by National Health Mission, Ministry of Health & Family Welfare Government of India

<https://nhm.gov.in/images/pdf/guidelines/nrhm-guidelines/stg/Hypertension_full.pdf>

8. Drug-Drug interaction and drug-disease will be assessed, and if harmful interaction occur, the prescription will be rated as harmful.

10. Unnecessary duplications of drug with the same indication will be rated as inappropriate.

11. For patient observation, prescription appropriateness will be rated pertaining to each main diagnosis given by the provider. For cases where no diagnosis was given, prescription will be rated based only on the drug’s dosage, frequency and duration to determine if it is harmful or not.
