# Supplementary material for: Barriers to effective hypertension management in rural Bihar, India: A cross-sectional, linked supply- and demand-side study
Source: PLOS Glob Public Health. 2022 Oct 12;2(10):e0000513. doi: 10.1371/journal.pgph.0000513 (PMC10021531; doi:10.1371/journal.pgph.0000513)
Supplement: S4 Annex — Provides a flow chart for the number of individuals and number of providers available at each level in the study, including the final number of hypertension patients linked to providers with known quality. The sample size of various regressions is also explained. (DOCX) [file pgph.0000513.s004.docx]

## S4 Annex: Linking process for determining percent of hypertensives linked to quality care


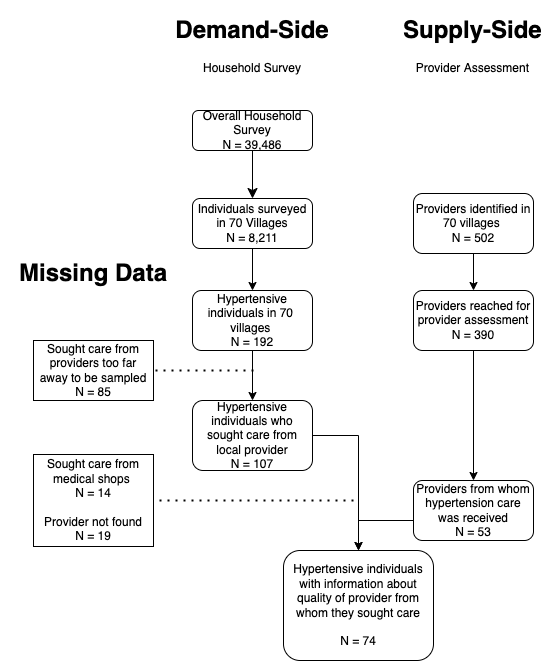


**Regression 1 sample size**: Hypertensive individuals with information about the quality of provider from whom they sought care. One individual from the ST caste reported being hypertensive, therefore this observation was dropped from the regression analysis to avoid an exposure that perfectly predicted the outcome. Final sample size is 73 individuals.

**Regression 2 sample size**: Bypassing among hypertensive individuals in the 70 randomly selected villages for provider quality assessment. This regression incorporates the number of providers of high quality in a village as a covariate. Final sample size is 192 individuals.

**Regression 3 sample size**: Bypassing among all hypertensive individuals. Final sample size is 950 individuals.
