# Supplementary material for: Barriers to effective hypertension management in rural Bihar, India: A cross-sectional, linked supply- and demand-side study
Source: PLOS Glob Public Health. 2022 Oct 12;2(10):e0000513. doi: 10.1371/journal.pgph.0000513 (PMC10021531; doi:10.1371/journal.pgph.0000513)
Supplement: S5 Annex — Provides a figure comparing the expanded hypertension care cascade between the Assessment of Primary Health Care in Bihar and the NFHS-4 studies. (DOCX) [file pgph.0000513.s005.docx]

## S5 Annex: Expanded hypertension cascade for adults age 30-54 in rural Bihar across two studies

*Legend: Bihar PHC = Bihar Assessment of Primary Health Care System study; NFHS 4 =National Family Health Survey 4 (2015-2016)*
