# Supplementary material for: Barriers to effective hypertension management in rural Bihar, India: A cross-sectional, linked supply- and demand-side study
Source: PLOS Glob Public Health. 2022 Oct 12;2(10):e0000513. doi: 10.1371/journal.pgph.0000513 (PMC10021531; doi:10.1371/journal.pgph.0000513)
Supplement: Attachment — Submitted filename: Reviewer Responses_April29.docx [file pgph.0000513.s007.docx]

Dear editors and reviewers,

Thank you for taking the time to review our manuscript. We appreciate the thoughtfulness with which you have approached this review and believe that the comments and revisions have made the manuscript stronger. Please find below a point-by-point response to all issues raised with our responses in red text.

Best,

Michael Peters, on behalf of the authorship team

Editors’ comments:

1. Your co-authors:
Yvonne Commodore-Mensah [-ycommod1@jhmi.edu](mailto:-ycommod1@jhmi.edu)
Japneet Kaur [-japneetkaur58@gmail.com](mailto:-japneetkaur58@gmail.com)
Navneet Kumar [-navneet.pcs@gmail.com](mailto:-navneet.pcs@gmail.com)
Krishna D Rao [-kdrao@jhu.edu](mailto:-kdrao@jhu.edu)
,have not confirmed authorship of the manuscript. We have resent them the authorship confirmation email; however please check that the above email address for them is correct and follow up personally to ensure they confirm.

Please note that we cannot proceed your manuscript  until we have received confirmations from all co-authors.

Thank you for this notification. I personally reached out on 4/13. Have confirmed with all co-authors.

2. Please provide  separate figure files in .tif or .eps format only and remove any figures embedded in your manuscript file.  Please ensure that all files are under our size limit of 20MB.

For more information about how to convert your figure files please see our guidelines: Once you've converted your files to .tif or .eps, please also make sure that your figures meet our format requirements:
<https://journals.plos.org/globalpublichealth/s/figures>

Thank you for this suggestion, it is done – newly uploaded files will be in .tiff form.

3. Please amend your detailed Financial Disclosure statement. This is published with the article, therefore should be completed in full sentences and contain the exact wording you wish to be published.
 
ii). State the initials, alongside each funding source, of each author to receive each grant.

Thank you for this suggestion. The following is the new Financial disclosure statement:

“KDR received funding through the Bill and Melinda Gates Foundation to conduct this research under grant number # OPP119434. The funder played no role in the design of the study nor the collection, analysis, and interpretation of data nor the writing of the manuscript”

4. In the online submission form, you indicated that "The data used in this study are owned by a research consortium, led by the Government of Bihar. Permission has not been given by the government to deposit the raw data in a public repository. The research consortium (and government) has reviewed and approved the publication of the results included in this study.

The datasets used and analyzed in the current study are available for individual use upon approval from the research consortium. All requests may be directed to the corresponding author.". All PLOS journals now require all data underlying the findings described in their manuscript to be freely available to other researchers, either 1. In a public repository, 2. Within the manuscript itself, or 3. Uploaded as supplementary information.

This policy applies to all data except where public deposition would breach compliance with the protocol approved by your research ethics board. If your data cannot be made publicly available for ethical or legal reasons (e.g., public availability would compromise patient privacy), please explain your reasons by return email and your exemption request will be escalated to the editor for approval. Your exemption request will be handled independently and will not hold up the peer review process, but will need to be resolved should your manuscript be accepted for publication. One of the Editorial team will then be in touch if there are any issues.

Thank you for this comment- we have sent the following email on April 29, 2022 to respond to this point:

Dear Editorial team,

Thank you for your comments on our article. The research consortium that sponsored this study was chaired by the Government of Bihar, and included Johns Hopkins, the Bill and Melinda Gates Foundation, CARE India, and Oxford Policy Management. Data collected through the study is the property of the Government of Bihar, and making the data publicly available will legally breach the understanding made between the Government of Bihar and consortium partners. While the Government of Bihar is fully aware and in support of the publication of these results, they have indicated that the raw data may not be made available for public access. This understanding was also included in the language of the research protocol approved by India’s SIGMA IRB, so making the data publicly available would also breach the protocol approved by the IRB. Therefore, we request an exemption to making the data underlying the findings in this article publicly available.

If you have any further questions or require additional documentation, we are happy to provide this. We are willing to elevate individual reasonable requests to the Government of Bihar to approve access to the raw data. Looking forward to your response.

Best,

Michael

Additional Editor Comments (if provided):

NA

Reviewers' comments:

Reviewer's Responses to Questions

**Comments to the Author**

1. Does this manuscript meet PLOS Global Public Health’s [publication criteria](https://nam02.safelinks.protection.outlook.com/?url=https%3A%2F%2Fjournals.plos.org%2Fglobalpublichealth%2Fs%2Fcriteria-for-publication&data=04%7C01%7Cmpeters%40jhu.edu%7C13b4413cc31b4c9355c908da167e88ce%7C9fa4f438b1e6473b803f86f8aedf0dec%7C0%7C0%7C637847029968120674%7CUnknown%7CTWFpbGZsb3d8eyJWIjoiMC4wLjAwMDAiLCJQIjoiV2luMzIiLCJBTiI6Ik1haWwiLCJXVCI6Mn0%3D%7C3000&sdata=KsG%2BlewYU7oMXBC528H6WVKhY3xO5Rc2ARrBCx6RX%2Bs%3D&reserved=0)? Is the manuscript technically sound, and do the data support the conclusions? The manuscript must describe methodologically and ethically rigorous research with conclusions that are appropriately drawn based on the data presented.

Reviewer #1: Yes

Reviewer #2: Yes

2. Has the statistical analysis been performed appropriately and rigorously?

Reviewer #1: I don't know

Reviewer #2: Yes

3. Have the authors made all data underlying the findings in their manuscript fully available (please refer to the Data Availability Statement at the start of the manuscript PDF file)?

The [PLOS Data policy](https://nam02.safelinks.protection.outlook.com/?url=https%3A%2F%2Fjournals.plos.org%2Fglobalpublichealth%2Fs%2Fdata-availability&data=04%7C01%7Cmpeters%40jhu.edu%7C13b4413cc31b4c9355c908da167e88ce%7C9fa4f438b1e6473b803f86f8aedf0dec%7C0%7C0%7C637847029968120674%7CUnknown%7CTWFpbGZsb3d8eyJWIjoiMC4wLjAwMDAiLCJQIjoiV2luMzIiLCJBTiI6Ik1haWwiLCJXVCI6Mn0%3D%7C3000&sdata=InUnCo%2FaFl3yBVc6mFi9LYcJmxO8ectRrAtrBtlfB9s%3D&reserved=0) requires authors to make all data underlying the findings described in their manuscript fully available without restriction, with rare exception. The data should be provided as part of the manuscript or its supporting information, or deposited to a public repository. For example, in addition to summary statistics, the data points behind means, medians and variance measures should be available. If there are restrictions on publicly sharing data—e.g. participant privacy or use of data from a third party—those must be specified.

Reviewer #1: Yes

Reviewer #2: No

4. Is the manuscript presented in an intelligible fashion and written in standard English?

PLOS Global Public Health does not copyedit accepted manuscripts, so the language in submitted articles must be clear, correct, and unambiguous. Any typographical or grammatical errors should be corrected at revision, so please note any specific errors here.

Reviewer #1: Yes

Reviewer #2: Yes

5. Review Comments to the Author

Please use the space provided to explain your answers to the questions above. You may also include additional comments for the author, including concerns about dual publication, research ethics, or publication ethics. (Please upload your review as an attachment if it exceeds 20,000 characters)

Reviewer #1: This research study describes the landscape of hypertension management in rural Bihar, India using linked supply- and demand-side estimates of quality care and receipt of care.

Thank you for your thorough review of our paper and for the constructive points you have raised- we believe that addressing your comments has strengthened the paper and look forward to your response.

Detailed comments:

Line 130: people who seek care for chronic conditions, 39% receive care from private MBBS and AYUSH providers, and another 30% seek care from IPs. What about the remaining?

Thank you, we added the following findings from the referenced study:

“Among people who seek care for chronic conditions in Bihar, 86% receive care from the private sector (39% from private MBBS and AYUSH providers, 30% from IPs, and 17% from pharmacists and other private sources), leaving only 14% who receive care from the public sector”

Line 219-221: “Measures of provider quality were described chronologically in terms of their impact on a patient’s progression through the hypertension care cascade, from screened to aware to treated and controlled BP.” As BP was not measured, not sure how the measure of BP control was evaluated.

Agreed – BP was not directly measured, so we have adjusted the language to indicate that we measure provider actions that contribute to patient ability to reach the next stage of the hypertension cascade (e.g., provider ability to diagnose hypertension contributes to the “aware” step in the cascade)

Edit: “Measures of provider quality were described chronologically in terms of their impact on a patient’s ability to progress through the hypertension care cascade, from screened to aware to treated and controlled BP. For example, a provider’s ability to appropriately diagnose hypertension based on a high blood pressure reading contributes to a patient’s ability to progress from “screened” to “aware”.”

Line 223: Definition of quality care needs to be revisited in the Indian context and provider type. The study investigators considered the criteria for “high quality HYPERTENSION care” to include availability of first-line antiHYPERTENSION medications. Majority of private doctors in India in single stand alone clinics do not stock antiHYPERTENSION medications.
In addition, in India, AYUSH providers are not legally allowed to prescribe allopathic medications and hence may not be stocking drugs.
IP are essentially unqualified medical providers- again not legally allowed to stock nor prescribe medications
These factors need to be highlighted in the manuscript and may have impacted the assessment.

Thank you for this insightful comment. We agree that the majority of private doctors in India do not stock antihypertension medications, however according to Indian guidelines on the management of hypertension, drug therapy should be started in individuals at the time of diagnosis. Further, there is global evidence that immediate initiation of antihypertensive treatment reduces adverse outcomes, suggesting that antihypertension medications should be readily available as a matter of best practice. We agree that we have not fully captured the nuance of the legality of AYUSH and IP providers and their ability to prescribe and stock these drugs. We believe that our definition of ability to deliver high quality hypertension care is still accurate, and have included the following in the discussion to fully capture this nuance:

“Additionally, the study definition of delivering quality hypertension care may have influenced results, as AYUSH providers and IPs are not legally allowed to stock or prescribe medications. Given the importance of immediate antihypertensive therapy in improving health outcomes, we believe that the availability of antihypertensive medications is an appropriate indicator of structural quality care.(37)”

Line 296: Gives breakup of health seeking behaviour of the population. However, the proportion seeking care from IPs is not clear. Of the 94.4% population who sought treatment from the private sector, how many sought treatment with IPs as they were the largest provider population assessed?

Thank you for raising this point - it is not possible to determine the qualifications of the provider from which care was received from the household survey. We asked individuals generally about the provider type they received care from (so we know they were private providers and not at PHCs) but we cannot expect patients to know the qualifications of their provider unless we follow up directly with the provider, which we do in the linked provider survey.

We do know the qualifications of private providers utilized in the 70 villages where the provider assessment took place (*linked assessment of hypertension management* section) and overwhelmingly, the majority sought care from IPs. We have added the following to indicate how many sought treatment from IPs in the case when provider qualifications are known:

“Of these individuals, 83.8% sought care from IPs, 8.1% sought care from private AYUSH doctors, 4.0% sought care from private MBBS doctors, and 4.0% sought care from public providers.”

Line 306: “Median monthly amount spent on HYPERTENSION management was 250 rs.” Please give the breakup by provider as the OOPE would vary.

Thank you for raising this point – as discussed in the previous comment, we can stratify by public and private providers, thus we have added the following:

“The median monthly spending is slightly higher for those who receive treatment from private providers than public providers (255 vs 200 rupees).”

Line 361: "...people from the general caste were significantly more likely to receive quality care from a local provider than those from the SC caste." What was the health seeking behaviour by caste? Were the SCs more likely to seek care from IPs/ AYUsh- hence impacting the quality of care received (as measured under the study)?

Thank you for this point – we think that the two are interrelated and agree that the differences in health seeking behavior should be emphasized. Added the following clarification:

“By caste, 93.3% of SC/ST individuals sought care from IPs compared with 80.0% of OBC and 85.7% of General caste individuals.”

Line 363: “Among individuals in the villages where quality of care was known, the number of quality providers in a village was not associated with the probability of bypassing local care options.” Not clear- please rephrase

Thank you for this feedback. We included another phrase to clarify this interpretation.

“In other words, there was no association between the number of local high-quality providers and the probability of bypassing local care.”

Line 395: “…the quality-adjusted coverage of hypertension management services was as low as 10.9%.” This statement should be clarified in context of local providers.

Thank you for this comment – we agree that we should condition this statement in the context of local providers and have edited the sentence to reflect this sentiment:

“While 95.7% of adults who were aware of their condition received some treatment, the quality-adjusted coverage of hypertension management services **by local providers** was as low as 10.9%.”

Line 396: “Nearly 45% of individuals with reported hypertension bypassed local providers, traveling between 20 and 40 kilometers to receive care.” This statement is not aligned with Line 424 that states: “over a quarter of individuals (28.2%) traveled more than 20 kilometers to receive treatment”

Thank you for pointing this out – we have aligned the two statements with the following revisions:

“Nearly half of individuals with reported hypertension bypassed local providers, traveling between 20 and 40 kilometers to receive care.”

“While most individuals with hypertension sought care from providers within five kilometers of their home (55.7%), many individuals also traveled long distances to receive treatment”

Line 444: “The capacity of all providers in rural Bihar, including IPs, to measure BP, diagnose hypertension, and stock and prescribe appropriate treatments must be improved.” Need to underline policy options, legal barriers and various challenges esp with regard to AYUSH and IPs.

Thank you, we agree with this point and have tried to account for nuance discussed as before with the following edits. We also have included options to expand pre-existing systems to expand access:

“The capacity of providers in rural Bihar, including IPs, to measure BP, diagnose hypertension, and stock and prescribe appropriate treatments must be improved within the existing legal constraints. Further, the role of community health workers, such as community health officers and accredited social health activists can be expanded in Bihar to bring services to populations.”

Line 462: Study concludes that patients may even suffer from adverse effects from the large proportion of providers who are unable to effectively provide hypertension management services. No data is presented on patients receiving harmful prescriptions that may cause adverse effects.

Thank you for raising this point. We think that this is an important finding that was removed from an earlier draft and have included the following sentences to address this:

“Among providers who correctly diagnosed the hypothetical patient as hypertensive, 16.6% of private MBBS doctors, 13.2% of private AYUSH doctors, 10.6% of IPs and, and 9.7% of public providers wrote prescriptions that were assessed to cause harm to the patient. Prescriptions were characterized as harmful because they included inappropriate drugs (e.g., Ampicillin, Betnisole, or Paracetemol), excessive doses, and/or harmful drug interactions.”

Other comments:
- IP- More background on informal providers (IPs) in the Indian context would be useful as they constituted more than 3/4ths of the total providers assessed

Thank you for this point – we completely agree and have added more information in the study context section:

“IPs are often trusted members from the community in which they serve, with limited health training or experience, charge nominal fees (generally 100 Rupees, or $1.37 USD per consultation), and are an important source of primary care services in rural Bihar and other parts of India, though their role has evolved differently in various market settings.(23) A recent nationwide study showed that in Bihar, 3.9% of villages had access to a public MBBS provider, 7.6% had access to any MBBS provider (public or private), and 96.2% of villages had access to any provider, including AYUSH or informal providers (16).”

- Annexes 1-4 - not available

Apologies for this omission– the submission guidelines for the journal did not allow for supplemental files in the first submission. These should now be available for your review

- Figure 1- not available

Apologies for this omission – the submission guidelines for the journal did not allow for additional files in the initial submission. These should now be available for your review

Reviewer #2: The authors have explored an interesting domain of NCD care (ie link between supply- and demand-side barriers) in rural Bihar. The manuscript has been prepared well. I have some minor queries and suggestions –

Thank you for your thorough review of our paper. We appreciate the time you took for your review and appreciate your comments on how to improve the manuscript. Please find below responses to your individual points:

1. Please avoid the term 'allopathy' for what we consider medical sciences as either 'evidence-based healthcare/ medicine' or 'modern medicine'. I would prefer the former term. The oft-used term allopathy creates confusion among both scientific readers and laypeople, as it continues the notion of error-prone healthcare practices which were prevalent during the time the word 'allopathy' was popularized by proponents of 'homeopathy'. Moreover, as far as I remember, the National Medical Commission doesn't use the term 'allopathy' either in its curriculum or degree. Similarly, the names of degrees for different steam of AYUSH should be mentioned somewhere (eg BHMS for homeopathy).

Thank you for this comment. We understand your concern and appreciate that there are multiple viewpoints on this topic. Understanding your perspective, we have made the following changes:

We have changed all instances of “allopathy” to “modern medicine”

We have included the following revision to incorporate degree names:

“The private sector includes (i) providers practicing modern medicine with a Bachelor of Medicine, Bachelor of Surgery, or MBBS degree, (ii) providers trained in Indian systems of medicine with a degree in Ayurveda (Bachelor of Ayurvedic Medicine and Surgery), Yoga and Naturopathy (Bachelor of Yoga), Unani (Bachelor of Unani Medicine and Surgery), Siddha (Bachelor of Siddha Medicine and Surgery), and Homeopathy (Bachelor of Homeopathic Medicine and Surgery), collectively known as AYUSH providers, and (iii) informally trained providers (IPs).(23)”

2. The authors have missed introducing the NPCDCS well in their introduction. They have not considered the presence of Population-based screening (PBS) for selected NCDs and their risk factors which includes hypertension too. The standard treatment guidelines mentioned at reference number 33 is not appropriate for use in building your justification for screening all adults 18 years and above (line number 387-388). This is in contrast to what they themselves have done while including only adults 30 years and older only. This age group is being screened according to guidelines mentioned in both NPCDCS modules and HWC (Health and Wellness Centre Modules).

Thank you for this comment. During stakeholder consultations, we found that the NPCDS was not very influential in Bihar, so we did not feel the need to go into great detail in the introduction. We agree that this is an important program in the national picture so have included the following:

“The National Programme for prevention and control of cancer, diabetes, cardiovascular diseases and stroke (NPCDCS) is the main program to address NCDs across India, and a recent review highlighted the need to strengthen public health facilities to provide screening, early diagnosis, and treatment.(16)”

Thank you also for suggesting additional resources to use for reference in screening guidelines. We would have liked to have asked the NCD module to all adults over 18, but time and logistical design prevented this. We have changed the citation and sentences to the following:

“According to NPCDCS and other screening guidelines, all adults over the age of 30 are supposed to have their BP measured opportunistically and during each interaction with the public health system”

Key stakeholders consulted during manuscript preparation suggested that the standard treatment guidelines, published by MoFHW, were appropriate for justifying screening procedures.

3. Though bypassing care provider is one of the key outcomes of the study, the same in missed in abstract's background/objective. Rather there is mention of supply- side barriers which I couldn't find sufficiently addressed in the introduction section or methods.

Thank you for bringing this point to attention. Supply-side barriers to care include considerations of quality of care, both structural and process-related. We have clarified this point using the following revision:

“This study describes population-level (demand-side) deficits in along the care cascade in Bihar, and gaps in the quality of hypertension care across provider types (supply-side) in India’s pluralistic health system”

We find bypassing of care to be the confluence of both supply- and demand-side barriers. While receiving high-quality hypertension care is the primary outcome of interest, the study is better powered to show the characteristics associated with bypassing care, which we take to be an important, but secondary measure of the study. We agree that the abstract is misleading and have removed the bypassing section from the abstract and replaced with findings on the probability of receiving quality care as follows:

“Individuals from the general caste with comorbidities living in villages with more high-quality providers were most likely to receive quality care from a local provider.”

4. There seems to be mismatch between proportion of respondents mentioned to have received quality of care (31.0% in line number 40 vs 10.9% in line number 43).

Thank you for pointing this out. These discrepancies come from the reporting from different studies. While 31% of all interviewed providers *could* provide quality care (i.e., demonstrated capacity), only 10.9% of patients actually received quality care (i.e., most patients went to low-quality providers). We have clarified with the following:

“Less than one third (31.0%) of all interviewed providers demonstrated ability to provide quality hypertension care”

“While 95.8% of diagnosed individuals received some treatment, only 10.9% of patients received care from quality local providers”

5. Though the objectives in abstract mentions intent to examine supply- and demand-side barriers, authors have mentioned mostly about demand side barriers in introduction (line numbers 114-118).

Thank you for this comment- we consider quality of care-related issues to be supply-side barriers to receiving effective hypertension treatment. We agree that this has not come out clearly, and have made the following adjustments to clarify this issue:

“This study describes population-level (demand-side) deficits in along the care cascade in Bihar, and gaps in the quality of hypertension care across provider types (supply-side) in India’s pluralistic health system”

6. Some of the references are inappropriate. For example, at line numbers 61-62, authors are arguing about hypertension being the leading CVD risk factor, but the references are unrelated mere narrative reviews or viewpoints and not scientific estimate studies or original articles. Similarly, I consider reference numbers 11, 16-18, 24, 32, 34 as either unrelated or inappropriate. In place of 24, identify a published reference from NSSO's most recent survey on health consumption pattern in India, since it has national-level estimates.

We appreciate your comment about your interpretation about the relevance of these references and appreciate the time taken to review each of these sources.

We have added a reference from the WHO Global Health Estimates and the Global Burden of Metabolic Risk Factors to address your first point. We believe that the viewpoints are necessary to cite to address the second half of the sentence, that providing hypertension control is necessary for achieving UHC, thus they are relevant.

Reference 11 is cited in the context of methods for calculating quality-adjusted coverage. This is the seminal paper in establishing effective coverage as the metric for monitoring UHC and how to measure it. The paper calls for more research on measuring effective coverage for diseases that match the context’s burden of disease, so we feel it is justified.

References 16-18 represent the published literature on Bihar-specific hypertension prevalence and treatment. We have removed these, but believe that the assertion can remain unchanged.

The existing reference 24 is an article about care-seeking specific to Bihar that is based on the NSSO’s most recent survey. Thus, we feel as though your concern is addressed and no further action is needed.

Reference 32 has been removed and replaced with the Prenissl study which demonstrates the hypertension cascade in Bihar.

We have changed reference 33 as stated in the response to comment 2

Reference 34 is necessary to demonstrate that self-reported hypertension status is a valid indicator of true hypertension status.

7. Authors have provided certain claims without any references. For example check line numbers 66-67, 76-77, 99-100, 163-164 (Demographic Health Service Provision Assessment Survey). Also, they should provide some reference for the ABPHC study/ report (line number 136).

Thank you for this helpful comment. For lines 66-67, we believe that the statement “health systems in LMICs have largely been unable to provide health services at sufficient coverage or quality to prevent deaths from NCDs” is a generally accepted fact among health systems researchers and is supported by evidence in the following sentence which is referenced. We have added the same reference after lines 66-67 to avoid confusion.

For lines 76-77, the hypertension care cascade is also well-known and supported by citations in the following sentence which demonstrate the use.

For lines 99-100, we agree that Bihar’s double burden of disease is not well known, so we have included the citation which comes in the following sentence (ICMR’s India State-level disease burden initiative) after this claim.

For lines 163-164, we agree that the SPA survey should be referenced and have added a new citation to the SPA methodology here (citation 26).

In line number 136, there is no reference available for the ABPHC study, as this is the first paper to be published out of the research effort.

8. I couldn't find reference number 12.

Reference 12 is under review at BMC Health Services Research. Since the first submission, a pre-print version has become available at the following link: <https://assets.researchsquare.com/files/rs-1183375/v1/014119e3-2e2c-44dc-aa6f-5e6b1d38d637.pdf?c=1647977962>. The citation has been updated accordingly.

9. Please correct few incorrect reference styles (eg 6, 7, 12).

Thank you for pointing this out – we have updated the reference styles on these citations.

10. The rationale is related to the concept of 'healthcare provider switch behaviour'. The authors are encouraged to add a few references to this phenomenon in their introduction to justify their research idea. They also need to build upon the case of 'poor coverage' of screening for hypertension through PBS of NPCDCS as well as poor health system preparedness found in the most recent National NCD Monitoring Survey. This is important since the tools used in NNMS were adapted from internationally standardized WHO-SARA and WHO-PEN tools as compared to those used by the authors.

Thank you for your comment. After a search of the literature, we could not find any relevant published articles that mentioned the concept of “healthcare provider switch behavior”. We have instead emphasized your suggestion in what we understand to be the closest theme to the proposed concepted through the following revision:

“This is significant because perceived quality of care is a major driver of provider choice, or switch, among Indian adults with hypertension.(18) This often leads to patients bypassing local care options to receive services that may be far away, but are more acceptable.(19)”

As stated in the article, the study tool was adapted from the internationally standardized WHO-SPA survey (which is preferred to the SARA survey since it provides sub-national estimates). The National NCD Monitoring Survey shows interesting national results, but estimates are not available at the state level. We agree that we can provide further evidence of the poor coverage of hypertension screening and treatment in Bihar, referencing the NFHS, which also uses the same internationally standardized questions as NNMS and WHO STEPS to construct the hypertension cascade of care. We have made the following revision to address this point:

“In Bihar, the coverage of screening and treatment services is low: less than half (46.8%) of hypertensive individuals were recently screened and aware of their high blood pressure status, and only 17.3% were receiving treatment.(18)”

11. I have not seen 'study context' being part of methods section. Some of the sentences here should better be part of introduction. Authors should tell a bit of health care system of Bihar to the international audience.

Thank you for this comment. The journal has allowed for this section to be placed in the methods section and we will defer to their judgement on the location of this section. We agree that we can introduce more of Bihar’s health care system and have added the following to the section:

“The health system is characterized by unequal access to health care, insufficient human resources and institutional capacity, poor quality care, and high out-of-pocket health expenditure: the state has the second highest ratio of private to public spending on health care in India.(21)”

“The public sector infrastructure includes about 1,800 Primary Health Centers (PHC), of which 455 are fully operational. PHCs are staffed by MBBS or AYUSH doctors, but weaknesses in the public sector caused by staff shortages and a lack of basic infrastructure and supplies further reinforce private health care-seeking behavior.”

12. I couldn't find the annexures, so I can't comment on them.

Apologies for this omission– the submission guidelines for the journal did not allow for supplemental files in the first submission. These should now be available for your review

13. The unit of study need to be clarified in study design part (line number 142-143). Is it individuals or households? If it is individuals, then mentioning use of three-stage sampling design is wrong.

Thank you for this point – individuals were the unit of study, but all individuals within selected households were included in the study (i.e., individuals were not a unit of sampling). To avoid confusion, we have clarified with the following:

“The ABPHC household survey employed a multi-stage sampling design”

14. The reason for 30 as cluster size is not mentioned (line number 146). The basis for sample size calculation is not provided too.

Thank you for your comment. We have noted that “The study’s sample size was designed to detect a difference in the proportion of patients who visit PHCs with and without competent clinicians”. This reflects the sample size motivation of the parent study.

Holding a constant “take size” from each cluster is important to minimize total variance for the clusters and reduce design effect. 30 units as a cluster size is well-recognized as a size that maximized survey efficiency and economy (WHO Epi cluster survey uses 30x30 cluster design). Please see articles on this topic such as <https://ete-online.biomedcentral.com/articles/10.1186/1742-7622-5-7> for additional details.

The specific sample size calculations for the study as referenced in the article is provided in Annex 1 which should now be available for review.

15. How was the questionnaire developed? How was it validated?

Thank you for raising this important question. We have included the following additional information about the questionnaire development and validation:

“Wherever possible, the household and provider assessment tools used validated questions from surveys implemented in the same context, including the National Family Health Survey, the National Sample Survey, and World Bank Health Care Provider Surveys.(15,26,27) The hypertension vignette was designed in consultation with cardiologists and pilot tested with local primary care providers in Bihar for psychometric validation.”

“The facility readiness assessment was modified from the validated Demographic Health Survey Service Provision Assessment survey, and was designed to understand the medicines, equipment, and human resources available to providers as compared to Bihar’s Essential Medicines List.(24,25)”

16. The authors have mentioned about use of 'trained enumerators'. If multiple data collectors were use, it would be appreciated to mention about the inter-observer reliability during or after training to decrease risk of measurement bias.

Thank you for this comment. Multiple data collectors were not used to collect information from the same household so inter-observer reliability is not calculable. However, measures such as supervisor-led interview observation, random spot checks, and logic checks through the CAPI software collection, we believe sufficient measures have been taken to decrease risk of measurement bias. We have added the following:

“Direct supervision and random response reliability checks were conducted in the field to ensure data quality and reduce risk of measurement bias”

17. Have the authors excluded specialist medical doctors (eg MD Medicine)? If yes, why?

Thank you for this point – there was no exclusion of specialist medical doctors, and specific details of providers’ medical degrees was obtained. The following has been added to specify the other types of medical degrees obtained:

“*Other allopathic trainings include degrees in dentistry and physiotherapy”*

18. I am not comfortable with the use of an unrelated facility readiness assessment tool for India. This is in contrast with what the NCD programme managers have been trained into.

Thank you for this comment. The facility readiness assessment tool was based off the internationally validated SPA survey, and adapted to the Bihar context based on the Essential Medicines List. The tool was validated by local partners and no issues were raised. Please see the following:

“The facility readiness assessment was modified from the validated Demographic Health Survey Service Provision Assessment survey, and was designed to understand the medicines, equipment, and human resources available to providers as compared to Bihar’s Essential Medicines List.(24,25)”

19. A flow-chart for different numbers for study participants at different stage of study would be appropriate.

Thank you for this suggestion – this is provided in annex 4, which should now be available for your review

20. Give full description for Stata software (College Station Texas). Is there a license for this software with any of the study team authors? I

Thank you for this suggestion. The license for this software is held by several of the study team authors but this is not usually written in manuscripts. We have edited to include the full software description and version number as follows:

“Data analysis were performed in StataSE version 14.1 (College Station Texas).(31)”
21. Check line number 320. Are AYUSH providers allowed to store medicines of modern medical sciences?

Thank you for pointing this out. In Bihar, AYUSH providers were often carrying pharmacological products. Since this has been pointed out as being against the law, we have removed this sentence from the paper and added the following to the limitation:

“Additionally, the study definition of delivering quality hypertension care may have influenced results, as AYUSH providers and IPs are not legally allowed to stock or prescribe medications. Given the importance of immediate antihypertensive therapy in improving health outcomes, we believe that the availability of antihypertensive medications is an appropriate indicator of quality care.(38)”

22. Check line numbers 348-349 and 359. In one place 74 people are mentioned to have their quality of care received known, while at other it is 73. Plz rectified this error.

Thank you for this comment. We have explained this discrepancy fully in Annex 4, and have also included the following footnote in the regression table:

“Of the 74 individuals whose quality of care was known, one individual from the ST caste reported being hypertensive, therefore this observation was dropped from the regression analysis to avoid an exposure that perfectly predicted the outcome. Final sample size for this regression is 73 individuals.”

23. Check line number 385-386. The national guideline is to screen for hypertension only once in a year. So why are you insisting on BP measurement at each visit to doctor?

Thank you for this comment. To be sure, we are not insisting on anything – we were referencing page 6 from the Standard Treatment Guidelines on Hypertension, published by the MoHFW, which states that “Screening for hypertension in all adults will involve opportunistic screening of people > 18 years of age at every point of contact with health professionals or allied health staff. This will include all points and levels of care – the village/multipurpose health worker, trained non-physician staff at sub-centres, primary health centre, community health centre, and referral hospital (District hospital, Medical College)”. Please also refer to the response given to your previous comment #2.

Further, in the NPCDCS operational guidance kindly suggested by you, BP measurement through “opportunistic screening” for hypertension is described as a separate approach to health camps, and are suggested for all individuals age 30 and above on visits to sub centres, PHC, CHC, and district hospitals (pg 9). Further, opportunistic screening for hypertension is recommended to occur during visits with AYUSH providers (section 1.4.7).

Therefore, we are insisting at BP measurement at each visit to a doctor for patients age 30 and older based on these two guidelines. We have adjusted as follows:

“According to NPCDCS and other screening guidelines, all adults over the age of 30 are supposed to have their BP measured opportunistically and during each interaction with the public health system”

24. Check line number 404-405, the claim that "local health systems are not providing adequate services to manage hypertension in Bihar" is ill-justified. The authors themselves have mentioned in limitation later that they couldn't contact the doctors outside the project villages who might have provided care to the study participants.

Thank you for this comment. We agree that we were not able to contact doctors that were located more than 5 km away from patients. Given the findings that local providers (those that were interviewed) could not provide high quality care, and that richer, more educated individuals are more likely to bypass local providers, we feel confident in saying that local health systems are not providing high-quality, equitable services to manage hypertension. We have adjusted the statement accordingly.

“In short, local health systems are not providing high-quality, equitable services to manage hypertension in rural Bihar.”

25. Check line numbers 396-97 and 424-25. There seem to be a typo or genuine error here?

Thank you for pointing this out – we have aligned the two statements with the following revisions:

“Nearly half of individuals with reported hypertension bypassed local providers, traveling between 20 and 40 kilometers to receive care.”

“While most individuals with hypertension sought care from providers within five kilometers of their home (55.7%), many individuals also traveled long distances to receive treatment”

26. Change the word 'biometric' with 'anthropometric' at line number 408.

Thank you for this suggestion – this change has been incorporated.

27. Choice of visit to 'urban areas' to seek healthcare should not be considered in isolation for hypertension diagnosis or refill of medicines as the authors are arguing. The healthcare seeking decisions (especially for ambulatory care) in this part of India is often taken to piggy-back with other important household need for travel to town. Since, the authors have not explored reasons for 'bypassing care' and traveling out of village, the justification is at best an educated guess.

Thank you for this point. We agree that people often travel to town for many reasons on top of receiving hypertension management. The justification comes from several discussions with household members that occurred during data collection, therefore we feel as though the hypothesis is stronger than an educated guess, as several respondents demonstrated their frustration with having to travel so far to receive quality care. We do appreciate your concern about this statement and have adjusted accordingly:

Removed the phrase: “implying that patients may be traveling to urban centers to seek care where the perceived quality of care is higher”

28. The role of CHOs (Community Health Officers) is not mentioned at all in hypertension screening, follow-up etc. This is surprising since the HWCs are being actively developed in almost all parts of India.

Thank you for this point – we were also surprised at the lack of the role of CHOs in Bihar. Our results suggest that community workers (and public options in general) are rarely utilized for care-seeking in Bihar. Less than 1% of hypertensive individuals interviewed received care from government clinics or community health workers. We have noted this omission in the discussion:

“Further, the role of community health workers, such as community health officers and accredited social health activists can be expanded in Bihar to bring quality services to populations”

6. PLOS authors have the option to publish the peer review history of their article ([what does this mean?](https://nam02.safelinks.protection.outlook.com/?url=https%3A%2F%2Fjournals.plos.org%2Fglobalpublichealth%2Fs%2Feditorial-and-peer-review-process%23loc-peer-review-history&data=04%7C01%7Cmpeters%40jhu.edu%7C13b4413cc31b4c9355c908da167e88ce%7C9fa4f438b1e6473b803f86f8aedf0dec%7C0%7C0%7C637847029968120674%7CUnknown%7CTWFpbGZsb3d8eyJWIjoiMC4wLjAwMDAiLCJQIjoiV2luMzIiLCJBTiI6Ik1haWwiLCJXVCI6Mn0%3D%7C3000&sdata=lAfYKO2dLhT8U938X4RjSZh%2FVEl3MiZhGI9qVeqUlj4%3D&reserved=0)). If published, this will include your full peer review and any attached files.


**Do you want your identity to be public for this peer review?** If you choose “no”, your identity will remain anonymous but your review may still be made public.


For information about this choice, including consent withdrawal, please see our [Privacy Policy](https://nam02.safelinks.protection.outlook.com/?url=https%3A%2F%2Fwww.plos.org%2Fprivacy-policy&data=04%7C01%7Cmpeters%40jhu.edu%7C13b4413cc31b4c9355c908da167e88ce%7C9fa4f438b1e6473b803f86f8aedf0dec%7C0%7C0%7C637847029968120674%7CUnknown%7CTWFpbGZsb3d8eyJWIjoiMC4wLjAwMDAiLCJQIjoiV2luMzIiLCJBTiI6Ik1haWwiLCJXVCI6Mn0%3D%7C3000&sdata=baLycA2Kf6re8os6kG1ZjNkUNUCfTxbxueb38GVT7q0%3D&reserved=0).

Reviewer #1: **Yes:** Anupam Khungar Pathni

Reviewer #2: **Yes:** Sanjeev Kumar
