# Supplementary material for: Barriers to effective hypertension management in rural Bihar, India: A cross-sectional, linked supply- and demand-side study
Source: PLOS Glob Public Health. 2022 Oct 12;2(10):e0000513. doi: 10.1371/journal.pgph.0000513 (PMC10021531; doi:10.1371/journal.pgph.0000513)
Supplement: Attachment — Submitted filename: Reviewer Responses_Aug8.docx [file pgph.0000513.s008.docx]

Line 174: “The public sector infrastructure includes about 1,800 Primary Health Centers (PHC), of which 455 are fully operational.” Please provide reference for the statement

Thank you for this comment. The 1,800 estimate comes from a Harvard report on financing in Bihar’s health system, which is now cited. The 455 number comes from internal CARE organizational documents, which is not public information. We have revised accordingly with the following statement:

“The public sector infrastructure includes about 1,800 Primary Health Centers (PHC), which represents about 60% of the PHCs required to serve Bihar’s population based national government estimates for infrastructure allocation.” (22)

2. Line 280: Please clarify what is implied by “initiated treatment upon diagnosis”. Does this refer to only medication as many patients with grade 1 hypertension may be started on lifestyle measures at diagnosis.

Thank you for this comment. We agree that it may be appropriate for a provider to only begin lifestyle advice. As noted in the discussion section, information on lifestyle recommendations during the vignette was collected, but such a small proportion of providers recommended lifestyle measures that we did not report on this. In the case of the vignette, the hypothetical patient had a blood pressure of 150/90, and this was the second reading of high blood pressure, meaning that it was appropriate for the provider to start medication.

Made the following change to this sentence: “Providers who made it to the final cascade step (had a functioning BP measurement device, would check a hypothetical patient’s BP at least once, made a correct hypertension diagnosis, initiated medical treatment upon diagnosis of stage two hypertension, had first line anti-hypertensive medication available, and wrote an appropriate prescription) are considered to deliver high quality hypertension care.”

3. Line 456: Hypertension prevalence is mentioned as 22%. Citation is incorrect to the data presented. May consider citing Geldsetzer et al.

Thank you for this comment and for catching the incorrect statistic. The hypertension prevalence in Bihar is estimated at 12.8% in the paper on India-specific hypertension awareness, treatment, and control by Prenissl et al (Geldsetzer is the senior author). We made the following change:

“Nearly one-quarter of adults in rural Bihar have never had their BP measured and just over eight percent of the adult population reported a previous hypertension diagnosis, compared to previous studies which reported a population prevalence of 12.8% in Bihar.(18)”

4. Line 473: “most individuals perceived the quality of care at PHCs to be poor”. Did the study assess the availability of staff at the PHCs that could have contributed to this perception?

Thank you for this question. We assessed numerous dimensions of quality of care including availability of staff, availability of medicines, and waiting/consultation times in the study. The statistic reported on in the paper refers to the question “why did you not seek care from your local PHC” when people self-reported bypassing the PHC to seek care. One response option was “low quality of care”, which was selected by 62.2% of people who bypassed the PHC to receive care. When asked the same question, only 11.5% of people bypassed the PHC to receive care because the doctor was not available. There are numerous dimensions of quality of care and this study is not designed to determine the drivers behind perceived quality of care, though this is an important area for future inquiry. We have emphasized a sentence in the discussion section:

“Future studies should seek to understand reasons for the perception of poor quality of care at public providers, even though PHCs often provide the highest quality of care locally available to patients.”

5. Line 481: Blood pressure measurement is not an anthropometric measure. Maybe the authors want to state “anthropometric measurements and BP”?

Thank you for this comment- we have included the following revision:

“First, the ABPHC household survey design was cross-sectional and did not include direct measurements of BP”
